# Dental Caries and Its Causes

**Published:** 1873-04

**Authors:** 


### BIBLIOGRAPHICAL.

*Dental Caries and its Causes.*—An Investigation into the Influence of Fungi in the Destruction of the Teeth. By Drs. Leber and Rottenstein. Translated by Thomas H. Chandler, D. D. S. Published by Lindsay & Blakiston, Philadelphia.

Some thirty years ago Dr. Bowditch, of Boston, published the result of certain investigations relating to the presence of animalculæ in the mouth, their influence upon the teeth, and also more directly in the formation of salivary calculus. Since, however, some European authors have professed to discover the presence of "fungi" in all diseased and fermenting matter, and the work under consideration is an advocate of the doctrine that such "fungi" produce dental caries.

The translator, however, does not appear to fully endorse such views as to the cause of caries of the teeth, but hopes that their presentation to the profession in this form may at least "stimulate investigation, and either by confirmation or disproval of its statements, advance the knowledge of the whole subject."

The work, which consists of one hundred and three pages, is illustrated by seven lithographs of sections of carious teeth, some of them magnified from one hundred to two hundred and fifty times.

